# Fragmented ambiguous objects: Stimuli with stable low-level features for object recognition tasks

**DOI:** 10.1371/journal.pone.0215306

**Published:** 2019-04-11

**Authors:** Cheryl A. Olman, Tori Espensen-Sturges, Isaac Muscanto, Julia M. Longenecker, Philip C. Burton, Andrea N. Grant, Scott R. Sponheim

**Affiliations:** 1 Department of Psychology, University of Minnesota, Minneapolis, Minnesota, United States of America; 2 Center for Magnetic Resonance Research, University of Minnesota, Minneapolis, Minnesota, United States of America; 3 Department of Psychiatry, University of Minnesota, Minneapolis, Minnesota, United States of America; 4 College of Liberal Arts, University of Minnesota, Minneapolis, Minnesota, United States of America; 5 Minneapolis VA Healthcare System, Minneapolis, Minnesota, United States of America; University of Pécs Medical School, HUNGARY

## Abstract

Visual object recognition is a complex skill that relies on the interaction of many spatially distinct and specialized visual areas in the human brain. One tool that can help us better understand these specializations and interactions is a set of visual stimuli that do not differ along low-level dimensions (e.g., orientation, contrast) but do differ along high-level dimensions, such as whether a real-world object can be detected. The present work creates a set of line segment-based images that are matched for luminance, contrast, and orientation distribution (both for single elements and for pair-wise combinations) but result in a range of object and non-object percepts. Image generation started with images of isolated objects taken from publicly available databases and then progressed through 3-stages: a computer algorithm generating 718 candidate images, expert observers selecting 217 for further consideration, and naïve observers performing final ratings. This process identified a set of 100 images that all have the same low-level properties but cover a range of recognizability (proportion of naïve observers (N = 120) who indicated that the stimulus “contained a known object”) and semantic stability (consistency across the categories of living, non-living/manipulable, and non-living/non-manipulable when the same observers named “known” objects). Stimuli are available at https://github.com/caolman/FAOT.git.

## Introduction

A dominant theme in visual neuroscience is that visual perception is accomplished by iterative computations in a hierarchical visual system that are modulated by behavioral goals and context. Thus, visual representations of objects are influenced by non-visual representations, such as task-dependent allocation of attention, which are computed in non-visual regions. Many studies indicate that visual computations are also altered by conditions such as psychosis [1, 2], autism [3], and aging [4], but it is not known exactly how these conditions affect neural mechanisms in the brain. We still need better methods for determining, in each case, whether the visual system is affected at a low level (retina, thalamus and primary visual cortex), an intermediate level (visual areas with retinotopic organization but selectivity for features of intermediate complexity [5]), a high level (object recognition regions), or in its ability to interact with non-visual brain regions. A quantitative approach to this problem requires careful control of visual stimulus features, since visual stimuli provide such a strong feed-forward drive to the brain that small changes to low-level features can have large effects that propagate throughout the system [6, 7].

Recent publications have demonstrated the need for image sets like the one we report here. For example, early fMRI studies of feedback processes in early visual processing revealed both positive [8, 9] and negative relationships [10] between early visual responses and perception of scene structure. Later, more detailed investigations indicate that enhancement and suppression of the early visual response, while subtle, depend on the complexity of the scene [11] and the spatial location in the scene [12]. At the other end of the spectrum, neuroimaging studies reveal relationships between frontal brain regions and visual regions during object perception [13, 14]. These kinds of studies are most powerful if low-level features do not co-vary with perceptual state.

The stimuli used in previous studies generally fall into three categories: 1) stimuli that do not change, but bistable perception or amodal completion results in spatial or temporal variation in perceptual state [10, 15]; 2) stimuli that only describe simple shapes that do not have a 3D, real-world interpretation [2, 8, 9]; and 3) stimuli that are more organic and familiar than the stimuli presented here, but vary in low-level features, either by the addition of line terminations or by the alteration of orientation distribution or the total number of line segments [10, 16]. Each of these approaches, while powerful, has its limitations. In the first case, relying on bistable perception limits questions to a narrow set of stimulus types. In the next case, we need stimuli that represent real-world objects, albeit in a simplified form, if we want to learn more about how the brain represents objects. Finally, using low-level feature changes to induce high-level perceptual changes will always raise the question of whether response differences arose in the early stages of visual processing.

The stimulus set developed in this study was inspired by those used by Cardin, Zeki and Friston [17] in a study of task-dependent functional connectivity between visual areas during a visual object recognition task. The stimuli from Cardin, et al., are similar to a set developed by Sassi et al [18, 19], which uses aligned Gabors to represent the silhouettes of figures from the Snodgrass and Vanderwart set [20]. However, the Cardin, et al., stimuli and the new set we describe differ importantly from the stimuli in Sassi, et al., in that we intentionally include ambiguous objects, which may be “recognized” by some participants but not others.

In the stimuli presented here, object and non-object percepts are readily suggested by clusters of line segments, embedded in a background of uniformly oriented line segments. Thus, images are equated for low-level features: every image has the same number of pixels that are white and the same number of pixels that are gray, avoiding response differences due to luminance [21, 22]; the total number of line segments in the image (object plus background) is always the same so contrast is equated [7]; orientation distribution is equated across image classes (meaningful and meaningless), eliminating possible differences due to representations of oriented edges in V1 [23]; every image class has the same number of line terminations, eliminating concerns about differential drive in primary visual cortex because V1 receptive field structure depends on stimulus sparseness [24]; meaningful and meaningless images also have the same probability of contours (defined by the distribution of orientation differences between neighboring elements), allowing quantification of object perception effects independent of contour detection effects, which are strong [8, 9, 11].

Because object perception is accomplished by iterative computations in a hierarchical visual system [25, 26], it is also useful to have a set of images that form a continuum between “object” and “non-object”, rather than simply occupy two categories. Stimuli that vary in ease of recognition, while still holding low-level features constant, will enable more nuanced studies of how object representations evolve during the 200–400 ms it takes to classify an object [14]. To accomplish the above goals, we have created a novel stimulus set that is intended to be broadly useful for elucidating the mechanisms of object perception.

## Methods

### Overview

The process of creating the Fragmented Ambiguous Object (FAO) images had three stages. Stage 1 was automated (creating Set 1); Stage 2 involved an initial rating of image utility by the authors (creating Set 2); Stage 3 involved recruiting external participants to engage in an object recognition task and analyzing rates of object recognition as well as the semantic content of naming responses to yield the final set of images.

### Participants

The 159 participants in Stage 3 (73 female, ages 18–59, mean age 45; mean IQ 105) provided written consent prior to participation in the study and all procedures were approved by the local Institutional Review Boards at both the University of Minnesota and the Minneapolis Veterans Administration Health Care System. All procedures were consistent with the Declaration of Helsinki.

### Stage 1: Creation of 718 candidate images

The original images used from this study were downloaded from the following publicly available object image databases:

Bitmap images from Appendix B of [27]: black and white line drawings
The Hatfield Image Test [28]: color photographs
Images for Psycholinguistic studies from http://leadserv.u-bourgogne.fr/bases/pictures/ [29]: black and white line drawings
The Bank of Standardized Stimuli (BOSS), https://sites.google.com/site/bosstimuli/ [30]: color photographs
The Relational and Item Specific Encoding Task http://cntracs.ucdavis.edu/rise [31]: color photographs selected from http://cvcl.mit.edu/MM/
Snodgrass and Vanderwart 'Like' Objects as described in [32]: black and white line drawings and full-color objects, available at https://figshare.com/articles/Snodgrass_Vanderwart_Like_Objects/3102781
International Picture Naming Project, https://crl.ucsd.edu/experiments/ipnp/ [33]: black and white line drawings

Our access and use of the images complied with the terms of service from all databases, which each stated that the images were made freely available to researchers. Three of the databases (Hatfield Image Test, BOSS, and the Snodgrass and Vanderwart ‘Like’ Objects) explicitly attached a Creative Commons license to their images; the others had no clear licensing statement other than to indicate that the images were made available free of copyright.

There is no broadly accepted standard for what types of objects should be included in a stimulus set. Previously published and influential studies of object recognition, such as [34] and [35], have worked with small and apparently arbitrary selections of objects. Many studies reference the influential work of Snodgrass and Vanderwart [20], who normed a set of 260 images on the basis of “familiarity” and “visual complexity”. The images in this set can be categorized as living/animate objects (insects, animals), living/inanimate objects (apples, celery), manipulable objects (candles, hairbrushes) and large/non-manipulable objects (buses, barns). Thus, the database selection for this study was governed by three criteria: we wanted (1) open access images, (2) roughly 2,000 images to start with, and (3) substantial numbers of both large and small, living and non-living objects.

Code for doing the following manipulations in Stage 1 was written in Matlab (MathWorks, https://www.mathworks.com/products/matlab.html) and is available on request. The goals of this first stage were to (1) render each object as a set of lines on a regularly spaced grid, to remove low-level feature differences, and (2) use a low-resolution process that would generate ambiguous or incomplete representations of the objects, so that some would be rendered unrecognizable. While other studies have worked to maintain careful fidelity to object boundaries (e.g., [18]) so all objects are recognizable, the following process intentionally rendered some objects so they were not recognizable.

Each image was 384x384 pixels and contained a hexagonal grid of 7-pixel line segments with center-to-center spacing of 16 pixels in the horizontal direction and 14 pixels in the vertical direction. Stimuli were presented with a subtense of 10°, resulting in line elements subtending 0.18° of visual angle and separated by 0.42°. The ratio of element spacing to element size (~2:1) is typical for stimuli used to study contour integration (e.g., [36]). The absolute size of the elements is on the small end of the normal range but was necessary to create objects that participants could perceive while fixating at the center of the image.

To generate a large number of candidate stimuli, images from the databases were scaled so the longest dimension was 336 pixels, to provide a border (equal to 6% of the image width on all sides) around the edge of each final stimulus image. Any color images were then converted to gray scale by averaging the Red, Green and Blue pixel values.

**Fig 1:**
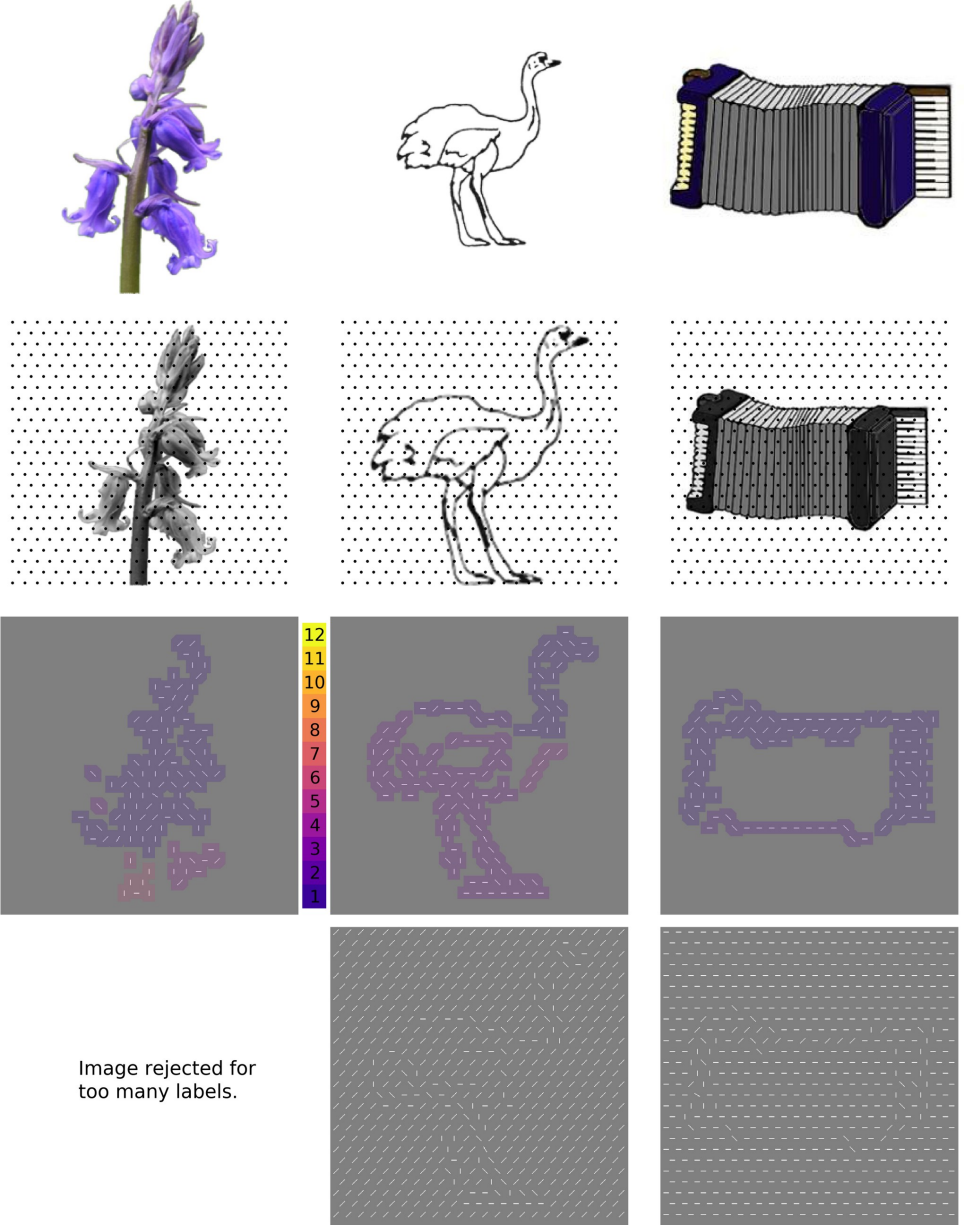
The first (automated) stage of stimulus generation. *Top row*: Line drawings, photographs, and full-color drawings of isolated objects were downloaded from existing object databases intended for vision reearch. *Second row*: Objects were scaled to occupy 88% of a 384x384 pixel square, then an algorithm using a regular grid of Gabor wavelet filters (dots indicate centers of Gabor wavelets) tuned to a range of spatial frequencies and orientations was used to convert local features to oriented line segments. *Third row*: the resulting array of line segments was convolved with a 16x16 pixel square, then subjected to a clustering algorithm that assigned a different integer to each disconnected region. “Objects” with 6 or more separate regions were eliminated from further consideration *Bottom row*: Each collection of object “features” was then embedded in a background of line segments at a randomly selected orientation. Credits, from left: bluebell_ed.bmp from http://testbed.herts.ac.uk/HIT/hit_apply.asp, shared free of copyright as stated in {Adlington}; 159.gif and Accordion.jpg from {Rossion} are downloaded from https://figshare.com/articles/Snodgrass_Vanderwart_Like_Objects/3102781, used with permission from Michael Tarr, original copyright CC BY NC SA 3.0 2016.

Filtering in the Fourier domain was used to estimate power in 8 different orientation bands (0°, 22.5°, 45°… 157.5°) for spatial frequencies between 24 and 120 cycles per image (3–15 cycles/degree). Then, a winner-take-all algorithm selected the orientation with the most power at each location on a regular, hexagonally close-packed grid (approximately 25 x 25 locations per image; 635 locations total, indicated by dots in the second row of Fig 1). Thus, images were converted to feature lists, which indicated the orientation of a line to be drawn at every location in which the original image contained part of an object.

Each feature list was then used to create a stimulus image in the following way. First, the underlying image was represented by placing line segments at the locations and orientations specified by the feature list. Next, a background line orientation was chosen at random from 8 possible orientations (0°, 22.5°, 45°… 157.5°) and the grid locations that did not contain part of the underlying object were filled in by lines at this background orientation. Pixels forming lines had a value of 255; the rest of the pixels in the image were assigned a value of 128. This process created 1,953 possible stimuli.

Next, an algorithm went through the potential stimuli and eliminated all stimuli that either had a small number of features (fewer than 80 out of 635 possible line segments in the feature list) or were disjointed. The threshold of 80 was chosen by visual inspection. Disjointed images were defined as having more than 5 separate labeled regions after applying Matlab’s *bwlabel* algorithm to a blurred version of the potential stimulus (Fig 1, third row). These incomplete images occurred frequently because the grid on which features were calculated could easily miss the edges that actually represented object boundaries in the images. The result of Stage 1 was 718 images that were potentially useful as stimuli.

### Stage 2: Determining the 217 images that would be viewed by naïve observers

The goals of this stage were (1) to reduce the number of objects to roughly 200, which was the largest sample size that could feasibly be viewed by a large population of naïve participants, and (2) to eliminate objects that had been camouflaged by the addition of background lines (i.e., which were disjointed once the background lines were added, which would not be detected by the algorithm that was written to eliminate disjointed images). The rationale for having a stimulus set of approximately 200 images is as follows. The images were to be included in a study recruiting roughly 150 participants; we expected good data from about 75% of the participants. Therefore, roughly 112 people would be providing ratings, and fatigue would decrease rating quality if each person were asked to rate more than 100 images (randomly picked from the total set). Therefore, to ensure at least 50 ratings per image, we wanted no more than 224 images (112 participants viewing 100/224 images = 50 ratings per image).

Laboratory staff (n = 4) then participated in a spatial two-alternative forced-choice experiment in which they viewed, on each trial, two of the 718 candidate stimuli and answered the question “Which image looks more like a single object?” All were aware of how the images were generated and how we planned to use them in visual studies. We explicitly did not judge objects based on whether or not they “looked like something”, since the aim was to cultivate a pool of images with a range of recognizability. Two stimuli were drawn for each trial at random, without replacement, resulting in 359 trials for a single session. Each observer completed four sessions, for a total of 1436 pairings per observer. Each image was therefore assessed in 16 comparisons.

**Fig 2:**
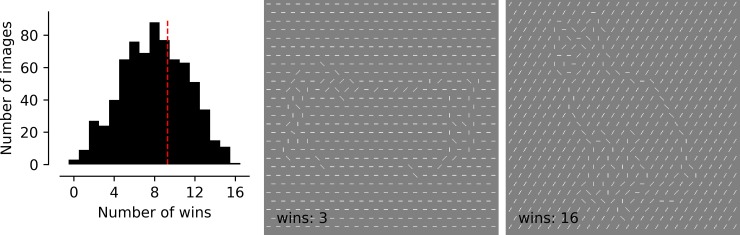
Histogram of number of wins for each of the 718 images ranked by authors. On the right are exemplars of the one image that won all 16 possible comparisons (cat, far right), and one of the many images that won only 9 out of 16 times and therefore was eliminated (the accordion from Fig 1, middle panel). The number of images included for consideration after this stage was 217.

Images were then ranked by the number of times they were selected in the 2AFC “Which looks more like a single object?” comparison by the authors, and the top third were selected for further consideration (Fig 2, cut-off = 9 wins). These were manually sorted to remove redundancies (e.g., several image databases include an airplane, but only one airplane was kept in the final set), and the end result was 217 images to include in Stage 3. This process intentionally excluded many images that could have been useful stimuli (i.e., images that were judged superior in more than 50% of their parings. In that sense, the selection of stimuli for the next stage was arbitrary (i.e., unbiased). However, it was necessary to reduce the number of stimuli for Stage 3, and the primary objective of this stage was to eliminate disastrously camouflaged objects, such as the accordion in Fig 2.

The 217 images that survived Stage 3 were characterized in four ways. The *orientation distribution* of line segments was computed, as well as the *distribution of relative orientations* between immediately adjacent line segments (a relative orientation of 0° indicates collinearity, so this statistic was computed to ensure that contours were not more frequent in recognizable images than unrecognizable images). The relative *density* of each “object” was computed by taking the ratio of the number of elements within the (irregular and concave) perimeter of the object that differed from the background orientation against the total number of grid locations within the object. Finally, *convexity* was scored because observers demonstrate a bias toward identifying convex features as belonging to the foreground under conditions of high uncertainty [37, 38], and we wanted to eliminate this possible confound from the final image set. The *convexity* of the object was scored by drawing a convex boundary around all grid locations that belonged to the object (i.e., differed from background orientation), and the “convexity” was the actual area of the object divided by the convex boundary around the object.

**Fig 3:**
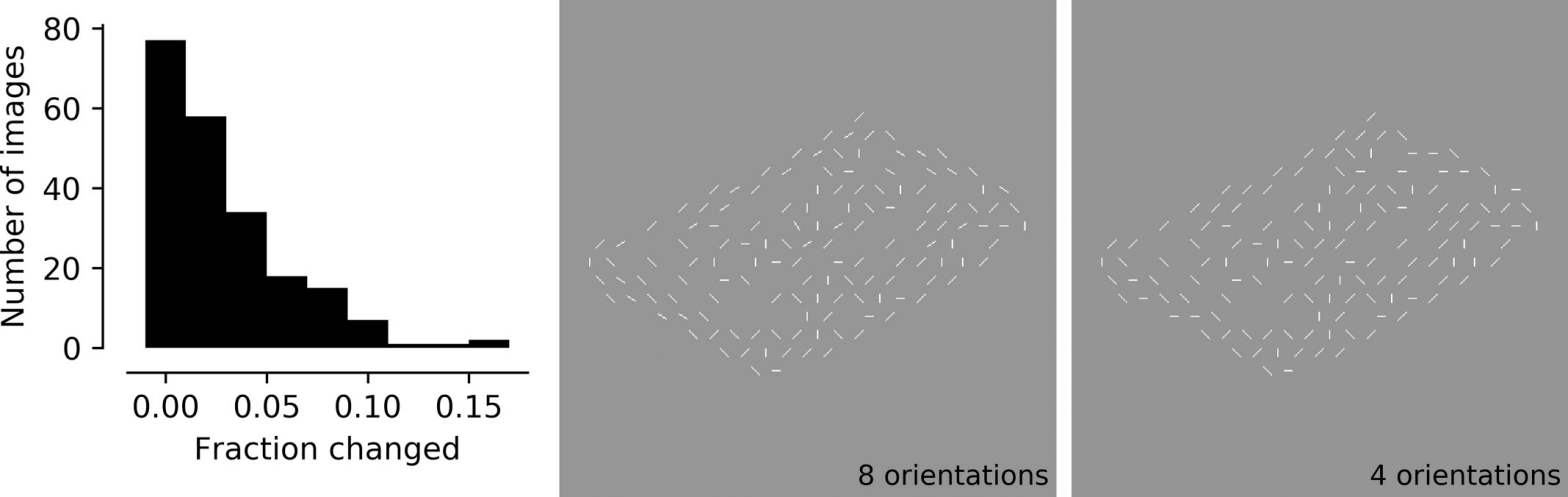
After the stimulus set was created, rare lines at orientations other than 0°, 45°, 90° or 135°were replaced. Left: in the majority of the images, fewer than 5% of the line segments were at 22.5°, 67.5°, 112.5° or 157.5°. These were therefore removed to simplify the final stimulus set. While observer ratings were performed on the images before the rare orientations were removed (8 orientations, middle panel), the changes were minor enough they would not be expected to change ratings by affecting perception of the object as a whole. See text for details.

Even though the features could theoretically take on one of 8 orientations, very few line segments (<1%) were actually assigned to orientations of 22.5°, 67.5°, 112.5° and 157.5° degrees. These rare non-cardinal, non-diagonal elements were included in the images viewed by naïve observers in Stage 3 (below), but they have been replaced by diagonal elements to simplify the final stimulus set. Therefore, the final stimulus set of 100 images contains line segments with only 4 orientations: 0°, 45°, 90° or 135°. Fig 3 shows a histogram of the fraction of the lines changed in each image (the distribution in the 217 images included in Stage 3 matches the distribution of the 100 images selected at the end of Stage 3) and an example of a stimulus before (8 orientations) and after (4 orientations) the changes were made. This example is one of the few images that had more than 10% of the lines changed; it is evident that the small and infrequent changes had negligible impact on the appearance of the stimulus.

### Stage 3: Responses from external volunteers

The 217 images generated by the above procedure were used in a “Yes/No” task asking: Does this image contain a known object? Images were displayed for a maximum of 7 seconds with a subtense of 10 degrees at a viewing distance of 133 cm; luminance of the gray background was 60 cd/m^2^. The trial ended when a participant used one of two buttons to respond either “yes” or “no” to the question “Does this image contain a known object?” If a participant did not answer “yes” or “no” within 7 seconds, that trial was discarded and not scored. Out of 217 possible images, each participant (N = 159) saw 100 randomly selected images. Therefore, images were presented an average of 73 times (159*100/217). Data were discarded for participants who responded “yes” more than 95% of the time or less than 5% of the time (presuming this reflected apathy toward the task and/or response bias); data were also discarded for participants who responded to fewer than 80% of the images (because each trial timed out after 7 seconds). Finally, data participants with Snellen acuity measured at 20/40 or worse on the data of the data collection were excluded, because visual acuity has been shown to affect contour performance [39]. Responses from the remaining 120 individuals were then used to calculate the probability that each image would be marked as a known object.

Because we wanted to ensure that this stimulus set was accessible to (resulted in object-like perceptions for) diverse participants, including psychiatric patients, this task was included in a family study of the effects of psychosis on visual perception similar to [40]. This study recruited broadly from the local community, advertising in clinics, on Craiglist and at local community centers. It could be argued that including patients with disorders known to result in abnormal visual perception as part of the “norming” population could bias the norms if these patients are more or less likely to mark an ambiguous image as recognizable. However, we also argue that it is important to include a diverse set of individuals in the rating stage, to ensure that the images are accessible to all and useful in studies of aging, autism and psychosis. An ANOVA testing for an effect of diagnostic group on recognition rate amongst the five groups (28 controls, 27 patients with schizophrenia, 24 patients with bipolar disorder, 21 relatives of patients with schizophrenia, and 20 relatives of patients with bipolar disorder) indicated no effect of group difference on recognition rate, F(4, 115) = 2.162, p = .078 (Table 1).

**Table 1:** Endorsement rates for groups participating in Stage 3. Endorsement rate is the fraction of images to which a participant responded “Yes, there is a known object”. Group (N): mean (standard deviation).

Group	Endorsement rate
Control (28)	0.37 (0.15)
Schizophrenia patients (27)	0.39 (0.16)
Bipolar patients (24)	0.51 (0.21)
Relatives of schizophrenia patients (21)	0.44 (0.22)
Relatives of bipolar patients (20)	0.42 (0.16)

The same external participants then completed a second task (immediately following the first) using the same images. This was a “Naming” task: every image to which they responded “yes” in the Yes/No task (and only the images to which they responded yes) was displayed a second time, for an unlimited amount of time, while participants typed in a name for the “object” they perceived. Participants also had the option of just hitting “enter” to refuse to label the image if, on further consideration, they could not come up with a name for the object. Of the 120 participants, the median participant said Yes to 40 images and named 28 images; 18 participants named 3 or fewer images; 4 participants named 75 or more.

All of the labels generated by participants were then categorized into one of 14 minor categories, which were then grouped into 3 major categories: living (minor categories: “human”, “animal”, “humanoid”, “instrument”, “food”, “plant”); non-living/manipulable (“object”, “weapon”, “body part”, “furniture”); non-living/non-manipulable (e.g., “building”, “place”, “vehicle”, “weather”). Categories were based on review of the literature [41] as well a collection of references reporting selective deficits for naming different object categories, e.g., body parts [42], food [43, 44] or animals [45]. The living category would ideally be broken down into two categories–animate and inanimate. However, the collection of images we used had few exemplars of food or plants that survived Stages 1 and 2, so we used a single top-level “living” category instead of sub-categories, as was done for non-living objects.

Using a combination of the Yes/No and Naming data, each image was scored for “recognizability” and “stability”. Recognizability, also referred to as recognition rate, was the proportion of responses, across 120 participants, that were “yes”. Stability was quantified by first determining the most common semantic category for an image then computing what fraction of labels assigned to the image belonged to the semantic category. For example, an image that was labeled “whale”, “stairs”, “entryway”, “whale”, “fish”, “cucumber”, “slide” would have a relatively low stability of 0.43 because the dominant category (living/animate) was only assigned 3 out of 7 times. An image that was labeled “goldfish”, “tennis racket”, “tennis racket”, “key”, “club”, “tennis racket” would have a high stability (0.83) because labels belonged to the dominant category (“non-living/manipulable”) 5 out of 6 times.

## Results

**Fig 4:**
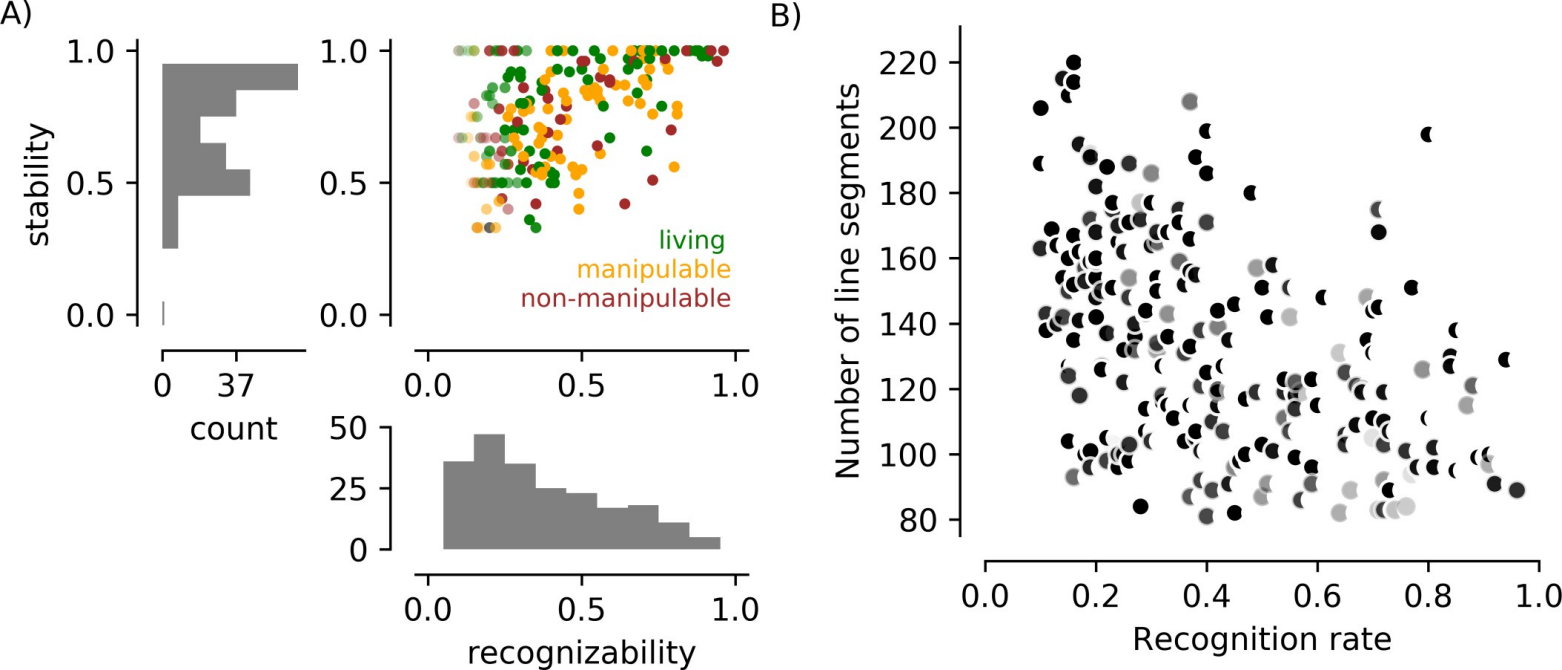
Behavioral responses and line segment counts for all 217 images in a study with naïve participants. A) The scatter plot shows the recognizability and stability scores for each of the 217 images, with the color of the dot indicating the dominant category for the image. The saturation of points representing images named fewer than 10 times is reduced. The black dot in the lower left indicates an image that was indicated as “recognizable” by 14% of observers in the Yes/No task, but then consistently rejected (by refusal to label) in the Naming task. B) The number of line segments used to describe each object was associated with recognition rates in this image set. The shape of the symbol reflects the convexity score: the more crescent-shaped, the smaller the ratio of the area of the object and the area of a convex bounding region. The darkness of the symbol reflects the density of the object, with black indicating objects that were defined by texture and light gray indicating objects that were defined by contours.

Fig 4A summarizes the responses by 120 external observers to the 217 images presented in the Yes/No and Naming tasks. In general, recognizability was associated with stability (see below for statistical assessment of this association in the final set of 100 images). Objects perceived as both living and non-living are found at the high and low end of recognizability and stability. No image received a score lower than 0.1 or higher than 0.9 on recognizability. On the high end, this is presumably because of finger errors or lapses in attention. On the low end, this is presumably because observers can idiosyncratically recognize objects in even the messiest scenes (like seeing shapes in clouds).

In Fig 4B, we show the association between the number of line segments in an image and the likelihood that it was deemed recognizable by a participant. A large number of line segments was associated with a low probability of recognition (*r = -0*.*44*, *p<1 x 10*^*−5*^). Recognition was also negatively associated with the density of the elements forming the object (r = -0.24, p< 1 x 10–3), indicating that objects with isolated contours were more easily recognized, and convexity (r = -0.18, p = 0.007), indicating that more convoluted objects were more difficult to perceive.

**Fig 5:**
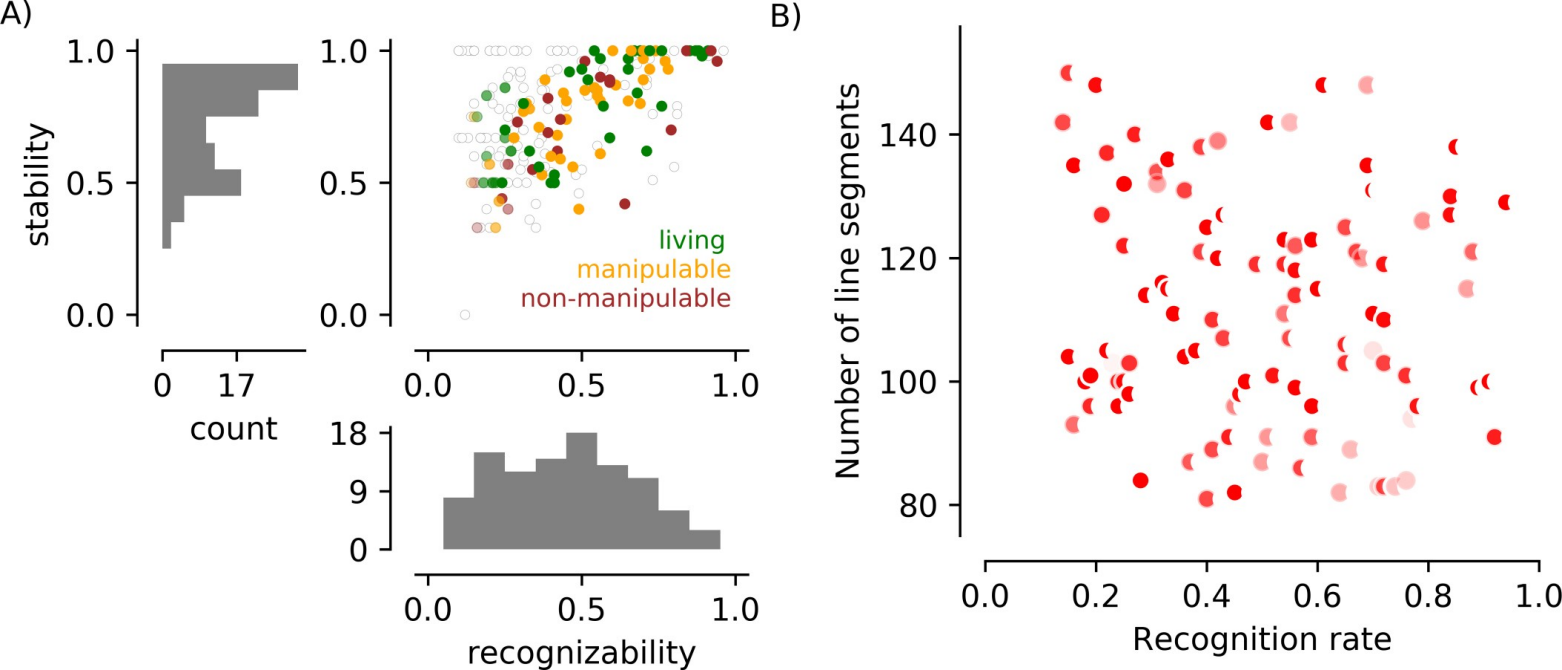
Behavioral responses and line segment counts for the subset of 100 images retained to create the vetted stimulus set. A) Scatter plot and marginal histograms as in Fig 4. Filled circles indicate stimuli included in the final set of 100 images; empty circles indicate stimuli present in Fig 4 but excluded from the final set. Median recognizability in the final set is 0.51 (mean = 0.51); median stability is 0.82 (mean = 0.77). In the final set of objects there are 40 living and 60 non-living (22 non-manipulable). B) Line segment number is not associated with recognition rate in the final set of 100 stimuli.

Selection of the final set of 100 FAO images, based on the data in Fig 4, had three goals: eliminate the association between number of line segments and object recognizability; create a set of images that were evenly distributed between “recognizable” and “not recognizable”; maintain variability in “stability”. This selection was accomplished by (1) discarding images in which more than 150 elements “belonged” to the image (66 images) or more than 10% were adjusted when non-cardinal orientations were removed (11 images), (2) discarding the rarely recognized (recognition < 0.5) but highly stable (stability = 1.0) images (12 additional images, represented by the left side of the line of dots across the top of the scatter plot), and (3) retaining 78% of the remaining images, with a bias toward images that were more often recognized but not highly stable (81% of images, or 50/62, with recognition rate > 0.5; 75% of images, or 50/66, with recognition rate < = 0.5). Recognizability and stability for this final subset of 100 images is plotted in Fig 5 (and tabulated with the dataset that can be downloaded at https://github.com/caolman/FAOT.git). This selection process successfully removed the association between number of line segments and recognizability (Fig 5B; r(98) = -0.14, p = 0.22), and reduced the associations with line segment density (r(98) = -0.19, p = 0.05) and object convexity (r(98) = -0.21, p = 0.04). Recognizability remained associated with stability (r(98) = 0.72, p<0.001). Recognizability and stability were not associated with semantic category (F(2, 97) = 1.19, p = 0.31 and F(2, 97) = 0.88, p = 0.42, respectively). Table 2 tabulates the various parameters that contribute to image complexity (number of line segments, density, convexity) for each object category in the 217 images that were used in Stage 3 and the 100 images selected after Stage 3.

**Table 2:** Mean recognizability, stability, density, and convexity, by category, for the 217 images that entered Stage 3 and the 100 images selected by Stage 3. For each statistic, mean (standard deviation) is shown.

Original 217	# images	Recognizability	Stability	nLines	Density	Convexity
living	88	0.42 (0.23)	0.80 (0.20)	130 (30)	0.95 (0.08)	0.84 (0.11)
manipulable	78	0.44 (0.20)	0.72 (0.18)	132 (34)	0.92 (0.11)	0.87 (0.11)
non-manipulable	49	0.42 (0.25)	0.75 (0.20)	139 (32)	0.97 (0.05)	0.89 (0.10)
**Final 100**						
living	40	0.51 (0.23)	0.79 (0.19)	110 (16)	0.94 (0.08)	0.84 (0.10)
manipulable	38	0.48 (0.18)	0.76 (0.18)	113 (22)	0.89 (0.14)	0.87 (0.10)
non-manipulable	22	0.51 (0.24)	0.73 (0.22)	114 (16)	0.96 (0.07)	0.88 (0.08)

**Fig 6:**
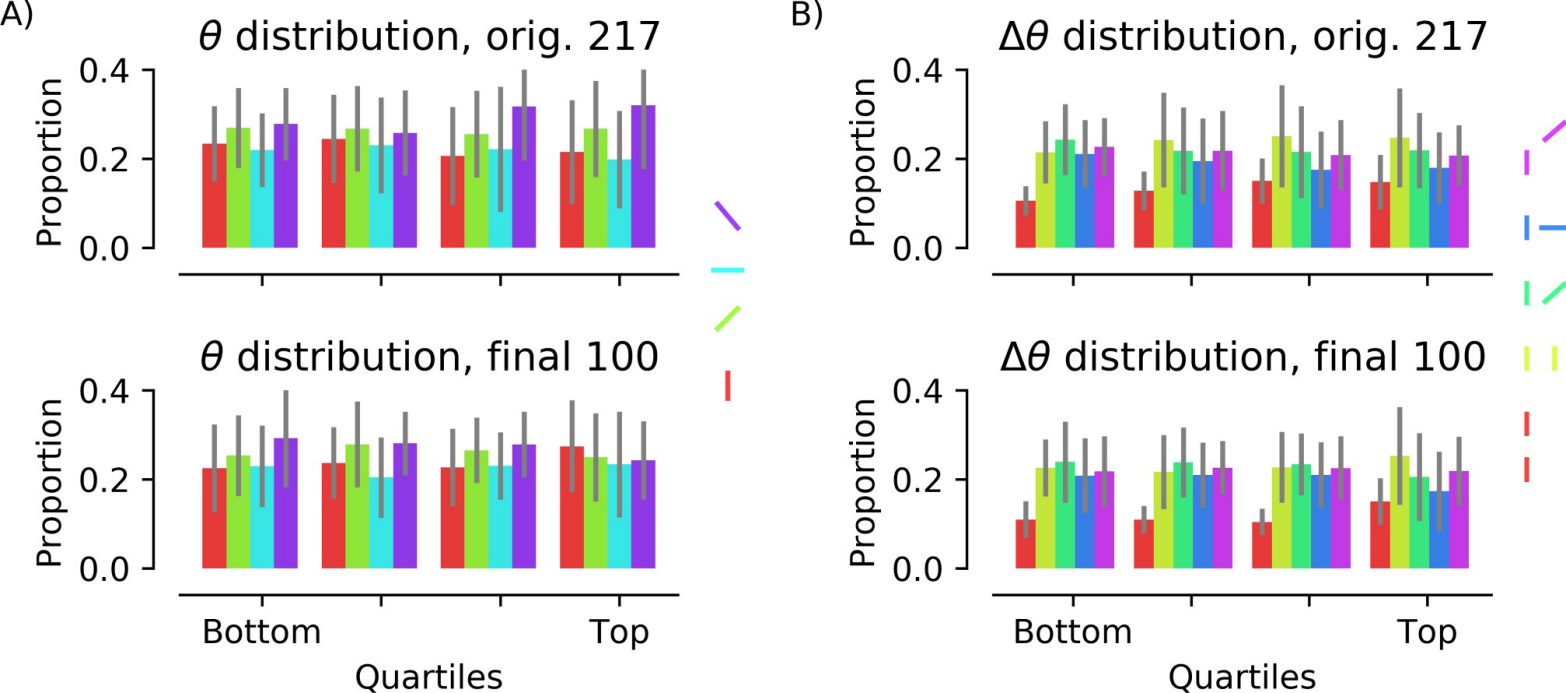
Orientations of line segments in intermediate and final image sets. A) Color indicates orientation: red, 0°; green, 45°; cyan, 90°; purple, 135°. Images were divided into quartiles based on the proportion of participants who indicated they contained a known object (“recognizability”). The orientations of line segments were not uniformly distributed but there was no correlation between the proportion of line segments at a given orientation and object recognition, r(98) = -0.11, -0.11, 0.092, and 0.10, respectively, for 0°, 45°, 90°, and 135°, all p’s > 0.2. B) Orientation difference between neighboring elements (red: collinear; yellow: parallel; green: acute; blue: orthogonal; purple: obtuse). There was also no correlation between the proportion of stimuli with a given pairwise orientation and object recognition for collinear, r(98) = 0.15, p = 0.14, parallel, r(98) = 0.12, p = 0.25, acute, r(98) = -0.06, p = 0.58, or obtuse, r(98) = -0.11, p = 0.29. The relationship between the proportion of orthogonal pairs and recognizability was the strongest, r(98) = -0.193, p = 0.055.

To further ensure that low-level feature bias was absent in the intermediate and final image sets, the orientation distribution of the line segments supporting figure/ground segmentation was calculated (Fig 6). (Overall luminance and contrast did not need to be computed because the method of stimulus creation made it impossible for these to vary.) In all quartiles of the intermediate image set and across the final image set there is a weak bias toward oblique line segments, likely because many of the objects in the image databases are “posed” at an oblique angle, but this is the same for “recognizable” and “unrecognizable” images. To determine whether collinearity of line segments predicted recognition, the distributions of relative orientations of neighboring segments were also computed (Fig 6B). Again, the distribution of relative orientations is stable across quartiles, indicating that collinearity of neighboring segments does not predict whether an object is recognized.

## Discussion

This novel stimulus set is useful for studying object recognition processes while keeping low-level visual information constant (i.e., independent of feature grouping or semantic content). The full package, available at https://github.umn.edu/caolman/FAOT.git, includes 100 fractured ambiguous object stimuli, documents and images indicating statistics of the stimuli and source images, and PsychoPy [46] code for presenting two tasks: the Yes/No task and the Naming task.

The “Gaborized outlines” stimulus set developed by Sassi et al [18, 19] is similar to this one in that it uses simple elements to represent complex objects with Gabor arrays that are relatively well matched for low-level features. As in our study, the Gaborized outline study explicitly separated detection and naming, citing a rich literature indicating that, except when stimuli are followed by masking stimuli [47], detection occurs more quickly and for a larger number of stimuli than naming [35, 48, 49]. A primary difference between their approach and ours is that we did not include an explicit representation of contours. In our new image set, the stimuli occupy a continuum between entirely contours and entirely texture, enabling the study of how different individuals use these different kinds of information, since bxwoth texture segmentation and contour perception contribute to object detection [18].

Our approach to the task design was also driven by concerns that clinical populations can be disproportionately impaired in even the phonological aspects of naming [50], as well as semantic processing to generate labels. While we did include images designed for psycholinguistic studies, our primary goal was to create an ambiguous set of stimuli, 50% of which were likely to generate object percepts and 50% of which were not. Therefore, we were careful to separate the object recognition (yes/no button press) and naming (typing) portions of the task. Even for work in neurologically typical populations, different neural networks and cognitive demands are associated with recognition versus naming, so the same stimulus set can be used to address a range of visual and cognitive processes.

The FAO images, and the stimuli in the other studies we have discussed thus far, do not attempt to match natural image statistics. By working with narrow-band stimuli like line segments and Gabor elements we are not providing the broadband spatial frequency content for which the visual system is optimized [7, 51]. For studies desiring to provide naturalistic stimuli, broadband approaches such as texture synthesis via a steerable pyramid of filters [52, 53] or image synthesis with filters learned via deep neural networks [54] provide good alternatives. However, in naturalistic stimuli it is more difficult to control local contrast, in particular the positioning of edges that provide such strong drive to early visual cortex. So while both types of studies are important, there is an important place in vision research for these impoverished stimulus sets that provide maximum control over low-level responses.

Images used in object recognition experiments are typically characterized by “visual complexity”; most commonly, complexity is rated by observers using a 5-point Likert scale, although on occasion, complexity is computed as a jpeg compression ratio [27] or by measuring features like edge density and convexity [55]. Because of the abstract structure of the images used here, and because many of the images did not look like objects to many of the observers, it did not make sense to ask observers to rate complexity. Low-, medium- and high-complexity images (as rated in previous studies) were included in the original images from which the final set of stimuli were derived. However, because the stimulus generation process manipulated the images so strongly, there was no association between the complexity of the original image and the recognizability of the final image. There was a weak and negative association between recognizability and both density and convexity (two factors that contribute to complexity) in the final stimulus set (Fig 5). Correlation between line segment density and recognizability was -0.19 (p = 0.05) and correlation between convexity and recognizability was -0.21 (p = 0.04). Neither correlation would be considered significant after correction for multiple comparisons, but this does indicate that less convex and less dense patterns are more likely to be recognized as objects.

One final and important characteristic of this stimulus set is that it is not intended to be used to characterize “normal” object perception, or perception of objects in the real world. The most obvious reason is that the representation of the objects is so sparse–contours are broken, jittered, and often absent. This means that there are not nearly as many cues for object identity in this stimulus set as in a normal object image set. Furthermore, the fractured nature of the stimuli, and the incompleteness of the bounding contours of objects, also requires participants to rely strongly on prior experience and mental imagery to perceive or name objects. Perception of objects in the real world does, almost always involves some kind of amodal completion (basically, filling in the gaps), because parts of objects often become occluded by other objects or the 3D nature of the object itself (self-occlusion). However, the amount of amodal completion required to form an object percept from a stimulus in this set is much higher than usual, making performance on tasks derived from this stimulus set more dependent on imagination or illusory perception than in other object image sets. Many of the existing object image databases also use “image variability”, which is measured by reaction time [29], to quantify the difference between the stimulus image and the mental image evoked by the stimulus. Image variability is obviously very high for the stimuli in this set because the images actually look nothing like objects. The Fractured Ambiguous Object set we present here is useful for studying object perception under conditions where (1) image information is low, (2) the contribution of amodal completion or even imagination is strong, and (3) image variability is extraordinarily high. These conditions do not mimic real-world object perception, but they do create an environment in which we can study how object detection or recognition performance depends on participant’s internal feature grouping and object recognition mechanisms when the image information is fractured and ambiguous.

## Conclusions

This work resulted in a set of 100 stimuli that can be used to probe object recognition while controlling the following low-level features: luminance, contrast, orientation distribution and number of elements contributing to potential object perception.
